# Block Sparse Compressed Sensing of Electroencephalogram (EEG) Signals by Exploiting Linear and Non-Linear Dependencies

**DOI:** 10.3390/s16020201

**Published:** 2016-02-05

**Authors:** Hesham Mahrous, Rabab Ward

**Affiliations:** ECE, University of British Colombia, 2332 Main Mall, Vancouver, BC V6T 1Z4, Canada; hesham.m.mahrous@gmail.com

**Keywords:** EEG signals, tele-monitoring, compressed sensing, BSBL, multivariate compression, linear and nonlinear dependency

## Abstract

This paper proposes a compressive sensing (CS) method for multi-channel electroencephalogram (EEG) signals in Wireless Body Area Network (WBAN) applications, where the battery life of sensors is limited. For the single EEG channel case, known as the single measurement vector (SMV) problem, the Block Sparse Bayesian Learning-BO (BSBL-BO) method has been shown to yield good results. This method exploits the block sparsity and the intra-correlation (*i.e.*, the linear dependency) within the measurement vector of a single channel. For the multichannel case, known as the multi-measurement vector (MMV) problem, the Spatio-Temporal Sparse Bayesian Learning (STSBL-EM) method has been proposed. This method learns the joint correlation structure in the multichannel signals by whitening the model in the temporal and the spatial domains. Our proposed method represents the multi-channels signal data as a vector that is constructed in a specific way, so that it has a better block sparsity structure than the conventional representation obtained by stacking the measurement vectors of the different channels. To reconstruct the multichannel EEG signals, we modify the parameters of the BSBL-BO algorithm, so that it can exploit not only the linear but also the non-linear dependency structures in a vector. The modified BSBL-BO is then applied on the vector with the better sparsity structure. The proposed method is shown to significantly outperform existing SMV and also MMV methods. It also shows significant lower compression errors even at high compression ratios such as 10:1 on three different datasets.

## 1. Introduction

For tele-monitoring and other applications involving EEG signals in Wireless Body Area Networks (WBANs) the amount of data acquired by the sensor is fairly large. Since the battery life of sensors in WBANs is limited, Compressed Sensing (CS) has been drawing much attention for such applications. CS requires less energy in compressing signals than existing techniques such as transform-based compression methods [1,2,3,4,5,6]. These CS methods have been applied to reconstruct single EEG channels [1,2,3,4,6]. The work in [2,3] proposes a CS framework for EEG signals. This framework is compared with those using the following dictionaries: a Gabor dictionary, a Mexican hat (second derivative of Gaussian function), a linear spline, a cubic spline, and a linear B spline and cubic B-spline. In [7], ECG and EEG signals were reconstructed using a linear B-spline wavelet dictionary and cubic B-spline matrix and reconstructed using Bayesian Compressive Sensing. The dictionary is randomly sampled and a modified banded Toeplitz dictionary matrix is formed. Another recent approach is to apply independent component analysis to pre-processes the EEG signals, prior to applying compressed sensing, so as to improve their sparse representation [8]. Zhang *et al.* have recently proposed reconstructed the EEG signals using a Block Sparse Bayesian Learning-Bounded Optimization (BSBL-BO) framework [1]. BSBL-BO reconstructs EEG signals without using a sparsifying dictionary matrix such as a Gabor dictionary. It is empirically shown to be highly effective in reconstructing EEG signals, as long as a low reconstruction error is tolerated. In [6], a compressive sensing framework is proposed where inter-channel redundancy removal is applied at the sensor after the sensing stage. Comparing the compression results of this approach with JPEG2000 and BSBL-BO shows that JPEG 2000 achieves the lowest error at high compression rates. However, the power consumption is of JPEG 2000 is too high and it is thus not suitable for WBAN applications.

The above studies have addressed the Single Measurement Vector (SMV) CS case *i.e.*, where single EEG channels are compressed and decompressed channel by channel. However, the simultaneous reconstruction of the CS signals from their multi-channel measurements (referred to as the MMV problem) has been shown to recover the signals more accurately than by applying SMV solutions on each channel separately [9,10,11,12]. In [11] the MFOCUSS algorithm extended the diversity minimization FOCUSS algorithm from an SMV to an MMV algorithm. MFOCUSS is an iterative re-weighted regularized algorithm that solves a l p norm minimization, where 0 < p ≤ 1 for MMV. In [10], the MFOCUSS was modified to tMFOCUSS by replacing the regularization with the Mahalanobis distance regularization, to capture the temporal correlation of the MMV signals. A similar idea is used in [9] to capture the temporal correlation, but in this case a Sparse Bayesian framework for MMV signals is employed. In [12] a STSBL-EM algorithm is proposed to solve the MMV model by exploiting the temporal and spatial correlation of the signals. It was to achieve somehow lower reconstruction errors compared to applying BSBL-BO on each channel. In this paper we propose a method to solve the MMV problem (and the SMV problem). We show that our proposed method outperforms existing algorithms in terms of reconstruction accuracy at different compression rates using three different datasets.

The previously mentioned work has focused only on reducing the power consumption of the data transmission part of the sensor, and only a few works have addressed the sensing and the processing power of the sensor node, however, the power consumption is still too high to consider in WBAN applications. Recent studies in [13,14,15] attempt to reduce the sensing and processing power. In [14] it is argued that to reduce the sensing energy, a smaller number of samples should be acquired directly in a random fashion. That is the signal should not be fully sampled and then compressed. Standard CS recovery techniques result in high NMSE so an alternative recovery approach based on the theory of low-rank completion is proposed. Their proposed algorithm is shown to be able to achieve power savings at a compression ratio of 2:1, the signal recovery is however poor at high compression ratios. In [15] it is shown that by applying blind compressed sensing; (a compressed sensing technique that involves dictionary learning) while solving the synthesis prior formulation, achieves better results. The work presented in [13] proposes a technique that combines blind compressed sensing with low-rank recovery (*i.e.*, combining the techniques in [14,15]). This technique achieved on average 0.18 NMSE for a compression ratio of 5:1, using the BCI data set in [16]. All of these techniques are not suitable for WBANs because the recovery quality is not high, hence high NMSE at high compression rates.

There are several studies that explain the physiological basis of EEG channels [17]. The EEG signals contain many redundancies that result in strong inter- and intradependencies within and among channels [17,18,19,20]. In this study, we estimate the linear dependency structure of EEG signals (by using the correlation measure) and the non-linear dependency (by using the phase locking values). We show the existence of linear and non-linear dependencies within and amongst the EEG channels. Unlike BSBL-BO [1] that exploits only the intra-block correlation structure (*i.e.*, linear dependence) to decompress the EEG signals, we modify the BSBL-BO so that it can exploit the linear and non-linear dependencies in EEG signals. The modified algorithm is shown to give better results than BSBL-BO and other existing algorithms, for the single channel (SMV) and the multi-channel (MMV) EEG problems. The modified algorithm reconstructs the multi-channels (MMV problem) by a specific vectorizing of the measurement vectors of the channels. We show that the DCT coefficients of this resulting vector (of the multi EEG channel signals) form a redundant block-sparse structure and that this structure has linear and nonlinear dependencies. This structure promotes low error even at high compression rates. We show that our proposed method (called BSBL-LNLD) outperforms many existing MMV methods and achieves a very low mean square error even at a high compression ratio of 10:1 (90% compression rate).

## 2. Background Literature

### 2.1. Compressed Sensing of L Dimensional Signals

Assume the number of EEG channels is L. For the *l*th channel, the corresponding CS model, denoted as the single measurement vectors (SMV) model is expressed as:

(1) $$y_{l}=Ax_{l}+v_{l}$$

In Equation (1), the vector $x_{l}\in R^{N}$ is the raw signal of the *l*th channel, $y_{l}\in R^{M}$ is the CS compressed signal, and $A\in R^{MXN}$ is the measurement matrix (also called the sensing matrix). $v_{l}\in R^{N}$ is the channel noise. N is the number of samples of $x_{l}$, and M is the number of samples after the reduction/compression of $x_{l}$. Traditional CS recovery algorithms use the compressed data $y_{l}$, and the sensing matrix *A* to recover the original signal $x_{l}$, the recovered signal is referred to as $\hat{x_{l}}$. The success of recovery relies on the key assumption that $x_{l}\;$ is sparse or has a sparse representation in a certain transform domain. When $x_{l}$ has a sparse representation in a certain domain, then $x_{l}$ can be expressed as $x_{l}=Dz_{l}$, where $z_{l}$ is the sparse representation of $x_{l}$ and $D\in R^{NXN}$ is a basis matrix that sparsifies the signal. For example D can be the discrete cosine transform (DCT) or the discrete wavelet transform (DWT) matrix.

Based on the sparsity requirements, to achieve the optimal reconstruction, Equation (1) is re-written as:

(2) $$y_{l}=ADz_{l}+v_{l}$$

Given *A* and *D*, a Compressed Sensing (CS) rule known as the Restricted Isometric Property (RIP) must be satisfied so that a perfect reconstruction is achieved at a minimum sampling rate given by [21,22,23,24]:

(3) $$M\geq {µ}^{2}\left(A,D\right).S.log\left(N\right)$$

Equation (3) shows the minimum value of M that can be chosen, so as perfect reconstruction is achieved. In Equation (3), S is the number of non-zero elements in $z_{l}$, and ${µ}^{2}$ is a coherence function between the two matrices A and D. The minimum value that can be chosen for M is dictated by S and ${µ}^{2}$. To achieve maximum incoherence, both matrices *A* and *D* should be selected carefully, so that D achieves a minimum *S* in sparsifying $x_{l}$. Much work has been done by previous researchers, to find the optimal D and A to achieve a minimum M. Unfortunately, finding the optimal D and A to compress any data or signal is not easy to achieve. Based on Equation (2), one may use a traditional CS algorithms to estimate $z_{l}$, and then calculate $\hat{x_{l}}$, that is:

(4) $$\min{\left\|\hat{z_{l}}\right\|}_{l1}\;subject\;to\;{\left\|AD\hat{z_{l}}-y_{l}\right\|}_{l2}$$

(5) $$\hat{x_{l}}=D\hat{z_{l}}$$

Equation (4) uses the Euclidian distance and minimizes the $l_{1}\;norm$ of $\hat{z_{l}}$. However, for highly correlated signals in the multivariate domain such as in EEG signals, the work presented in [9] shows that minimizing the Mahalanobis distance signal measurement achieves better results for highly correlated signals in the multivariate domain such as in EEG signals. The result of using the Mahalanobis distance is presented in Section 4.

### 2.2. Block Sparse Bayesian Learning via Bounded Optimization (BSBL-BO)

BSBL-BO is a CS framework [24] that has been recently proposed for solving the Single-Measurement-Vector (SMV) model (*i.e.*, the model Equation (1) with *L* = 1). While some CS algorithms depend on the sparsity of the signal, BSBL-BO exploits the block sparsity in the signal, provided the signal is block sparse [1]. That is, BSBL-BO assumes that the vector $x_{l}$ consists of (g non-overlapping) blocks and some of these blocks are all zeros. As mentioned in [1,24], the block size can be arbitrarily and the block partition does not need to be consistent with the true block structures of the signal.

Raw EEG signals generally do not have clear block structures in the time domain. Therefore, BSBL-BO is applied on the DCT coefficients of the signals [1]. By using the DCT dictionary matrix, an EEG signal is expressed as a DCT coefficient vector, where the coefficients with significant nonzero values concentrate at the lower frequencies of the coefficient vector (from the “energy compaction” property of DCT). The coefficient vector can be viewed as a concatenation of one or more nonzero blocks followed by a number of zero blocks. Therefore, BSBL-BO can exploit this specific block sparsity in the DCT coefficients by first obtaining the DCT coefficients and then reconstructing the original EEG signal.

The BSBL-BO algorithim is derived by applying type II maximum likelihood derivation [24] of the posterior probability given as:

(6) $$p\left(\hat{x}|y,\lambda ,{\left\{\gamma_{i},B_{i}\right\}}_{i=1}^{g}\right)=N\left(\mu_{x},\Sigma_{x}\right)$$

The hyperparameters λ, ${\left\{\gamma_{i},B_{i}\right\}}_{i=1}^{g}\;$ represent the noise (λ), the block sparsity structure ($\gamma_{i}$), and the intra-correlation structure ($B_{i}$) in the non-overlapping blocks g. Let $\Sigma_{0}$ be a diagonal matrix such that $\Sigma_{0}=diag\left({\left\{\gamma_{i}B_{i}\right\}}_{i=1}^{g}\right)$. After estimating the hyper-paramteres, the reconstructed signal $\hat{x}$ is estimated by minimizing the negative log-likelihood Equation (6). The resulting estimate of the reconstructed signal is given as: $\hat{x}=\mu_{x}=\Sigma_{0}A^{T}{\left(\lambda I+{A\Sigma }_{0}A^{T}\right)}^{-1}y$.

The hyperparameters $\lambda \text{ and }\gamma_{i}$ are derived based on the bound-optimization estimation in [24]. λ is a scalar that helps the algorithim perform in noisy conditions. In noiseless cases, λ is fixed to a small value, e.g., $\lambda =10e^{-10}\;but\;when$ the SNR is less than 15 dB, λ is estimated using the bounded optimization technique given in [24]. This yields $\lambda \leftarrow \frac{{\left|\left|\left(y-A\mu_{x}\right)\right|\right|}_{2}^{2}+\sum_{i=1}^{g}\text{Tr}\left(\Sigma_{x}^{i}{\left(A^{i}\right)}^{T}A^{i}\right)}{M}$. The hyperparameter $\gamma_{i}\;$ is a nonnegative scalar that controls the block-sparsity of the signal. When $\gamma_{i}$ = 0, the corresponding $\hat{x}$ of the $i^{th}$ block = 0. This hyperparaemter is given as $\gamma_{i}\leftarrow \sqrt{\frac{x_{i}^{T}B_{i}^{-1}x_{i}}{Tr\left({\left(A^{i}\right)}^{T}{\left(\Sigma_{y}^{*}\right)}^{-1}A^{i}B_{i}\right)}}$.

The other hyperparameter, $B_{i}\in \;R^{d_{i}\times d_{i}}$ is a positive definite matrix that captures the intracorrelation structure of the $i^{th}$ block. $d_{i}$ is the number of samples of the $i^{th}$ block. The intra-correlation is useful because it indicates a predictive relationship that can be exploited. Equation (7) below is a covariance matrix which is derived by minimizing the negative log-likelihood of the posterior probaility Equation (6). $B_{i}$ is further modified to obtain $\hat{B_{i}}$ Equation (8) by constraining it to be a positive definite intracorrealtion matrix. $\hat{B_{i}}$ is formed using a first-order Auto-Regressive (AR) process which is sufficient to model the intra-block correlation [24]. The resulting $\hat{B_{i}}$ is a Toeplitz matrix that is selected to represent the intra-block correlation matrx $B_{i}$:

(7) $$B_{i}=\frac{1}{g}\overset{g}{\underset{i=1}{\sum }}\frac{\Sigma_{x}^{i}+\mu_{x}^{i}{\left(\mu_{x}^{i}\right)}^{T}}{\gamma_{i}}$$

(8) $$\hat{B_{i}}=\text{Toeplitz}\left(\left[1,\bar{r},\ldots {\bar{r}}^{d_{i}-1}\right]\right)=\left[\begin{array}{ccc} 1 & \bar{r}\cdots & {\bar{r}}^{d_{i}-1}\\ \vdots & \ddots & \vdots \\ {\bar{r}}^{d_{i}-1} & {\bar{r}}^{d_{i}-2}\cdots & 1 \end{array}\right]\forall i$$

The first order Auto-Regressive coefficient is $\bar{r}=\frac{\bar{m_{1}}}{\bar{m_{0}}}$, where $\bar{m_{0\;}}$ & $\bar{m_{1}}$ are the average of the elements of the main diagonal and sub-diagonals of the estimated covariance matrix $B_{i}$. In BSBL-BO, $\hat{B_{i}}$ captures the intra-block correlation structure by converting the estimated covariance matrix $B_{i}$ to a bounded first order Toeplitz matrix. The intra-block correlation is a measure of linear dependency. In the next section, we modify the BSBL-BO so it can exploit both the linear dependency structure as well as the non-linear dependency structure in EEG signals.

## 3. Approach and Implementation

### 3.1. Approach

It has been shown that better reconstruction can be obtained by exploiting the block-sparsity (assuming the data vector is block sparse) than by only exploiting the sparsity in the signal (assuming the vector is sparse in the conventional sense) [1,25,26]. The conventional sparsity solution method only assumes that $x_{l}$ has at most S non-zero elements, in a sparse domain. However, it does not exploit any further structure that the signal may have. The non-zero components can appear anywhere in $x_{l}$, however, there are cases in which the non-zero values can form blocks [26]. We propose to apply the block-sparse recovery approach to the EEG multiple measurement vector (MMV) problem as the MMV data share a joint sparsity pattern [27,28,29]. For the case of EEG signals, the channels have linear and non-linear dependencies structure between them as well as within a channel [19,20,29].

**Figure 1:**
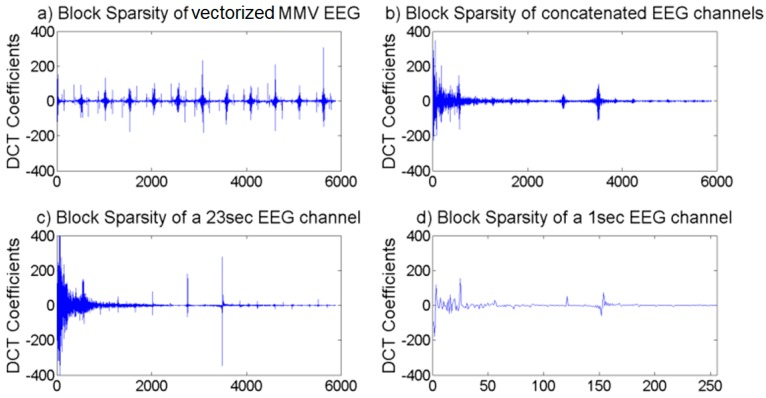
Block Sparsity of EEG DCT Coefficients of EEG channels. (**a**) The DCT coefficients of $vec\left[X^{T}\right]$; (**b**) The DCT coefficients of vec[X] ; (**c**) The DCT coefficients of $x_{l},\;when\;x_{l}$ is formed of 23 s of data of the channel *l*; (**d**) The DCT coefficients of $x_{l}$ when it is formed of one second of data of the same channel.

The work presented in [1] addresses the SMV case. It uses a DCT dictionary matrix (that results in energy compaction of a single EEG channel) to obtain a vector of block sparse DCT coefficients. These DCT coefficients are recovered by BSBL-BO in [1]. To study the MMV case, we first investigate the structure of the MMV data vector. For the MMV case let X be the matrix $\left[x_{1},x_{2},\;\ldots x_{L}\;\right]$ where L is the number of channels. In conventional studies vec[X] *i.e.*, the vector formed by stacking the columns of X has been studied. However in this paper we propose to study $vec\left[X^{T}\right]$ *i.e.*, the vector formed by stacking the rows of X as a vector. The DCT coefficients of $vec\left[X^{T}\right]$ and vec[X] are shown in Figure 1a,b, respectively. These correspond to the case when the number channels L is 23. The DCT coefficients of Figure 1c are the DCT transform of $x_{l},\;when\;x_{l}$ is formed of 23 s of data of the channel *l*, and the DCT coeficients of Figure 1d are the DCT transform of $x_{l}$ when it is formed of one second of data of the same channel. In Figure 1c, the length of the channel was formed of 23 s so as to result in the same number of coefficents as those of Figure 1a,b. This paper compresses the DCT coefficients of multi-channel EEG signals, which we denote as $D^{T}vec\left[X^{T}\right]$. $D^{T}vec\left[X^{T}\right]$ has more of a block sparse structure than the vector formed by concatenating the channels of the EEG signals, *i.e.*, $D^{T}vec\left[X\right]$. The blocks in $D^{T}vec\left[X^{T}\right]$ also have more structure than the DCT coefficients of a single channel which we denote as $D^{T}x_{l}$. Figure 1a shows that the MMV vector, $D^{T}vec\left[X^{T}\right]$, exhibits more structure and more redundant non-zero blocks than the vector formed by concatenating the channels, $D^{T}vec\left[X\right]$, (Figure 1b) and $D^{T}x_{l}$ (Figure 1c,d). This is investigated further in more details in Section 3.2.3, and Section 4.3.

BSBL-BO exploits the intra-block correlation, which is a measure of linear dependency in the blocks of a single channel data (temporal data only). Previous works however show that EEG signals and neurophysiological signals exhibit linear as well as non-linear dependencies [19,20]. In [19], EEG signals are examined for non-linear interdependence between channels, and a significant evidence of the existence of non-linear interdependency is shown. In order to describe the structure of EEG signals, the work in [20] suggests that nonlinear dependency measures, such as: mutual information, entropy, phase synchronization, and state space synchronization are not intended to substitute linear dependency measures such as auto regression (AR) and cross correlation. Instead, non-linear dependency must be regarded as a complement to the linear dependency when studying EEG signals. This allows a more comprehensive structure in the EEG data.

Based on our observation above, we therefore vectorize the signals of the multichannel data as $vec\left[X^{T}\right]$ *i.e.*, in a way that is different from the conventional one. This will help us better exploit the block-sparse structure of $vec\left[X^{T}\right]$ exhibited in Figure 1a. We also show that this resultant multichannel vector has significant linear and non-linear dependencies. In our method, the compressed data is reconstructed using this vectorization in conjunction with a modified version of BSBL-BO, which will be presented in Section 3.2.4. As will be shown, we will modify the matrix $\hat{B_{i}}$ Equation (8) in BSBL_BO. This matrix is Toeplitz and its AR coefficients model the intra-channel correlation for every channel by exploiting the intra-block correlation of each EEG channels. The modified version of $\hat{B_{i}}$ combines the Phase Locking Values (PLV) of the blocks (so as to exploit the non-linearity intra-dependence) with the intra-channel correlation. The use of $Dvec\left[X^{T}\right]$ instead of processing single channels, would enable the exploitation of the intra-blocks interdependencies and modelling the intra and inter dependencies (whether linear dependence, non-linear dependence, or both) of channels. Applying the modified BSBL-BO on the vector from the suggested vectorization ($vec\left[X^{T}\right]$ will enable us to exploit the linear and non-linear dependencies within the channels of the EEG data as well as between the channels. The detail of the modified algorithm follows in the next subsection.

### 3.2. Implementation

**Figure 2:**
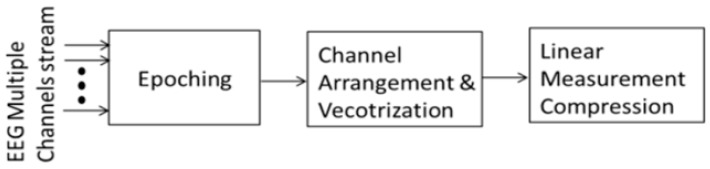
Block Diagram showing our approach for multivariate compression in CS.

The corresponding CS model for the L channels case, denoted as the MMV problem is expressed as Y=AX+V, where $Y=\left[y_{1},y_{2},\ldots y_{l}\right],\;X=\left[x_{1},x_{2},\ldots x_{l}\right]$, $V\;=\;\left[v_{1},v_{2},\ldots v_{l}\right]$, ($X\in R^{NXL},Y\in R^{MXL}$, $V\in R^{NXL}$), and N is the number of samples per epoch. In this paper, the matrix $X^{T}$ is vectorized so that the measurement vector is represented as $y=Avec\left[X^{T}\right]+v$. The implementation of the compression technique is shown in Figure 2 and implementation is discussed in the following subsections.

#### 3.2.1. Epoching

The EEG data of each channel is divided into non-overlapping epochs each of size *N*. In our experiments, we choose *N* to be equal to the sampling frequency of the dataset *i.e.*, it corresponds to one second. After the compression, the data of each epoch are recovered independently from other epochs.

#### 3.2.2. Channel Arrangement and Vectorization

In [1], BSBL-BO was developed to decompress a single vector and was thus applied on each SMV channel. To compress the multiple channels, in this paper the BSBL-BO in [1] is modified and then applied to reconstruct the channels jointly and exploits the linear and nonlinear dependencies of the EEG signals. Given a data matrix $X\in R^{\text{NXL}}$, whose columns are the data of the L channels then (as mentioned above) the matrix X is transformed into the vector $vec\left[X^{T}\right]={\left[x_{1,1},\;x_{2,1},\;\ldots x_{L,1},\;x_{1,2,}x_{2,2}\ldots \;x_{L,2}\ldots x_{1,N}\ldots \;x_{L,N}\right]}^{T}$, and also into the vector $vec\left[X\right]={\left[x_{1,1},\;x_{2,1},\;\ldots x_{N,1},\;x_{1,2,}x_{2,2}\ldots \;x_{N,2}\ldots x_{1,L}\ldots \;x_{N,L}\right]}^{T}$. When $vec\left[X^{T}\right]$ is divided into non-overlapping blocks, di's, such that $d_{i}>2L$ then each block of $vec\left[{\hat{X}}^{T}\right]$ would contain both temporal and spatial information about the data. It is thus important that $d_{i}>2L\;$ otherwise temporal correlation would be neglected.

**Figure 3:**
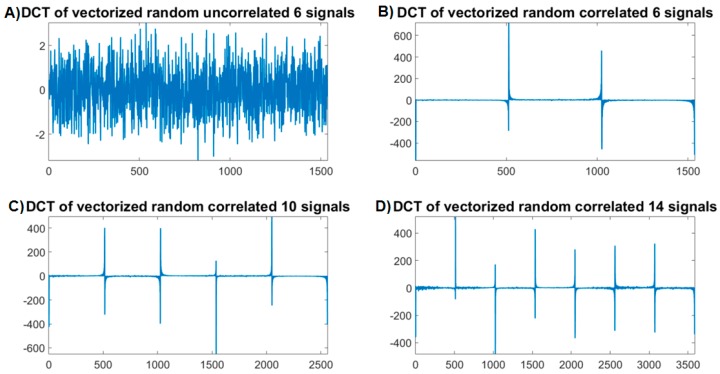
Block structure of correlated and uncorrelated signals in the DCT domain. (**a**) The DCT coefficients of the vectorized form of uncorrelated random signals; (**b**) The DCT coefficients of the vectorized forms of correlated 6 channel signals; (**c**) The DCT coefficients of the vectorized forms of correlated 10 channel signals; (**d**) The DCT coefficients of the vectorized forms of correlated 14 channel signals.

As shown in Figure 1a the DCT coefficients of $vec\left[X^{T}\right]$ exhibit better block sparsity than the DCT coefficents of vec[X] and of $x_{l}$ shown in Figure 1b–d. The structure shown in Figure 1a is found to be consistent for different data samples. The DCT of $vec\left[X^{T}\right]$ shows that this distinct block-sparse structure has a redundant form. In Figure 1a the non-zero values forms blocks that repeat in a consistant fashion. This structure does not exist for uncorrelated signals. To prove this emperically, the DCT of $vec\left[X^{T}\right]$ is examined when $X^{T}is\;formed\;of$ uncorrelated and of correlated random variables as show in in Figure 3a–d Figure 3a shows the DCT coefficients of the vectorized form of uncorrelated random signals. Figure 3b–d shows the DCT coefficients of the vectorized forms of correlated multichannel signals generated of random multi-channels variables.

As shown in Figure 3b–d, the DCT coefficients of the vectorized correlated signals exhibit a distinct block sparse-structure. The redundancy of the non-zero structure increases by increasing the number of channels. As the number of correlated channels increases the number of structured non-zero blocks increases and this increases the accuracy of the recovery for high compression rates. This is illustrated in Figure 3b–d.

#### 3.2.3. Compression

As mentioned in Section 2.1, the compression effectiveness depends on the degree of coherence between the matrices A and D. It is shown in [5,21,22] that the higher the incoherence between A and D, the larger the compression that can be achieved. This is valid only when the R.I.P condition applies. Regardless of the type of D, to achieve maximum incoherence, A should be independent and identically Gaussian distributed [5]. Using a Gaussian Ν$\left(0,\frac{1}{N}\right)$ results in an optimal sensing matrix [5], but the generation of such a matrix is computationally expensive. For energy saving purposes, a sparse binary matrix that contains few non-zeros (of value equal to one) in each column of the A matrix was used in [1,6]. It was shown that two non-zero entries are sufficient to compress the signals when the positions of the non-zeros entries are randomly selected in each column of A [1]. Also, it was shown that the sparse binary matrix performs as well as using a Gaussian matrix [6].

For the MMV problem, to compress $vec\left[X^{T}\right]\;$for every epoch, we use $A\in R^{LM\;X\;LN}$, where *M* is the number of random projections that determines the compression ratio given by N/M  (or the compression rate percentage $CR\%=\left(1-\frac{M}{N}\right)100$). L is the number of channels of the EEG signals. To solve the MMV problem, the compressed data is given by $y=A\;vec\left[X^{T}\right],\;\text{where}\;y\in R^{LM}$. In case of the SMV problem, $y_{l}=Ax_{l},\;\text{where}\;y_{l}\in R^{M}$ and $A\in R^{M\;X\;N}$. The matrix A is fixed for the measurement of all epoches.

#### 3.2.4. Modification of BSBL-BO (BSBL-LNLD)

As mentioned in Section 2.2, BSBL-BO exploits the block-sparse structure by learning the hyper-parameter $\gamma_{i}$. It exploits the intra-block correlation (in a single channel) by learning the hyper-parameter $\hat{B_{i}}$. The hyper-parameter $B_{i}$ is evaluated by minimizing the negative log-likelihood with respect to $B_{i}$ [24]. The resultant derivative is shown in Equation (7). $B_{i}$, is transformed to $\hat{B_{i}}$ by constraining it to being a positive definite and symmetric matrix. Assuming all the blocks have the same size, the idea is to find one parameter from which a close estimate of $B_{i}$ is formed. The $\hat{B_{i}}$ formed using the parameter, $\bar{r}$, is especially close to $B_{i}$ along the main diagonal and the main sub-diagonal of $B_{i}$ [24]. Further, it is found that for many modelling applications, if the elements of a block form a first-order Auto-Regressive (AR) process then this is sufficient to model the intra-block correlation [30]. In this case, the covariance matrix $B_{i}$ of the block (Equation (6)) is converted to a Toeplitz matrix $\hat{B_{i}}\;$ as shown in Equation (8). The parameter $\bar{r}$ is the AR coefficient. Instead of estimating $\bar{r}$ from the BSBL cost function, it is empirically calculated as $\bar{r}=\frac{m1}{m0}$. For most of the time m0 is greater than m1, which makes $\bar{r}<1$. If for any case $\bar{r}>1$, then $\bar{r}$ is constrained to be equal 0.99. This is done in order to insure that $B_{i}$ is always invertible.

To exploit the linear and nonlinear dependencies, we modify $\bar{r}$ so that it can also learn the non-linear dependency and not only the linear one, but the rest of the algorithm remains unchanged. The Phase Locking Value (PLV) between each block of $Dvec\left[{\hat{X}}^{T}\right]$ and every other block is calculated. Here $\hat{X}$ is a matrix of size N x L that represents the reconstructed signal at every learning iteration. The PLV between every two non-overlapping blocks is calculated and then averaged to become a scalar p. This scalar value, p, represents the average phase synchronization between all blocks. Since each block of $vec\left[{\hat{X}}^{T}\right]$ contains temporal and spatial information about the EEG signals, then p captures the intra and inter non-linear dependence in the EEG channels.

The PLV was proposed in [31] to measure the phase relationship between two neuro-electric or bio-magnetic signals. The PLV between two signals varies from 0 to 1. When the PLV between two signals is equal to one, this implies that the signals are perfectly synchronized. When the PLV between two signals is equal to zero this implies that the signals are completely out of synchronization. The PLV between two signals is given as:

(9) $$PLV=\frac{1}{N}\overset{N}{\underset{i=1}{\sum }}\text{exp}\left(j\left(\emptyset_{1,i}-\emptyset_{2,i}\right)\right)$$

where $\emptyset_{1,i}-\emptyset_{2,i}$ will now be explained: to obtain Equation (9), each signal is converted into the Hilbert space to obtain a real and imaginary part for each sample of the signal i. The phase angle $\emptyset_{1,i}$ and $\emptyset_{2,i}$ are then obtained by calculating arctan of the real and imaginary values. Thus $\emptyset_{1,i}-\emptyset_{2,i}$ is the phase angle difference between two signals in each sample. Phase synchronization is useful because the phase component is obtained separately from the amplitude component. Unlike coherence, it does not separate the effects of amplitude and phase in the interrelations between two signals.

To exploit the information about the phase synchronization in BSBL-BO, the parameter $\bar{r}$ is modified so that it learns the linear and also the non-linear dependencies in a vector. Thus when applied to vector $Dvec\left[{\hat{X}}^{T}\right]$ which contains the inter- and intra-information about the multi channels, $\bar{r}$ would learn both types of dependencies in the channels. As such $\bar{r}$ would contain information about inter relationships between channels and not only intra relationships in each channel. This would then allow BSBL-BO to exploit the inter- and intra linear/non-linear dependence in the channels instead of the linear dependency (intra-correlation) only. The modified $\bar{r}$ is given as $\bar{r}=\left(\frac{m1}{m0}+p\right)/2$, where p is the average of the PLV between the blocks. In the experiments section we show the performance of the modified version, BSBL-LNLD, in a compressed sensing framework for both SMV and MMV problems (we denote our modified algorithm as BSBL-LNLD).

## 4. Experiments and Results

### 4.1. Data Set

The experiments were conducted on three datasets. Dataset 1 is the BCI Competition IV [16]. This dataset is recorded from seven healthy subjects. The recording was made using 59 EEG channels per subject at a sampling rate of 1000 Hz. Dataset 2 is a collection of 108 polysomnographic recordings of different sleep disorders monitoring. Each recording contains between five and 13 EEG channels sampled at 256 Hz [32]. The third dataset consists of seizure data of 22 pediatric subjects. The recordings contain 23 EEG channels at a sampling rate of 256 Hz [33].

### 4.2. Dependence Measure of Intra and Inter EEG Blocks

In this experiment, we study the correlation and the PLV measures over the same data samples. A total of 3000 samples were used, 1000 samples from each dataset. Each sample of the EEG data was selected randomly from a dataset and was over a one second window of the multiple channels. Each sample was thus formed of the data of all channels in that dataset, each sample was transformed into its DCT coefficients and the vectors, $D^{T}vec\left[X^{T}\right],\;D^{T}vec\left[X\right],\;\&\;D^{T}x_{l}$ were formed. Each of these three vectors was divided into equal size blocks, then the intrablock and interblock correlations and PLV were calculated for each vector. Then the absolute value of these measures were averaged over all 1000 samples taken from the dataset it belongs to this experiment was then repeated few times but for different block sizes. That is each sample was again divided into equal size blocks but for a different block size. The results of correlation and PLV of different block sizes are shown in Figure 3. Correlation is a measure of linear dependency and it ranges between −1 and 1. When the correlation measure is positive, then the average block correlation is a positive correlation. When the correlation measure is negative, then it is a negative correlation. The phase synchronization measures such as the PLV finds a statistical relationship between two signals and varies from 0 to 1. PLV is important because EEG signals are noisy and chaotic, thus two or more EEG signals may have their phases synchronized even if their amplitudes are uncorrelated over time [20].

**Figure 4:**
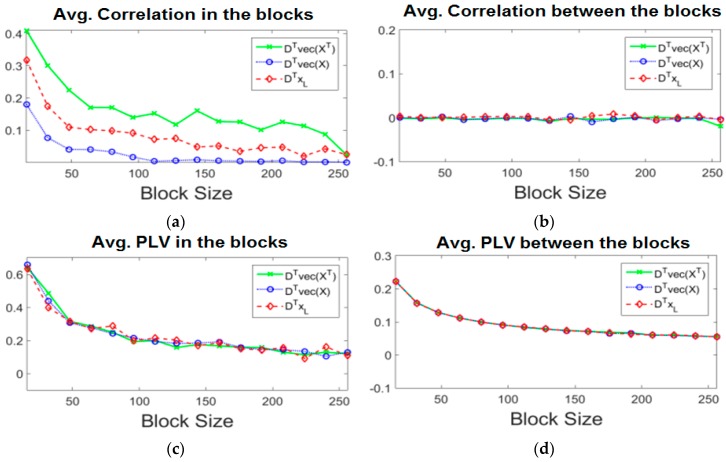
Mean Correlation and PLV in the blocks and between the blocks. (**a**) Average Correlation in the blocks; (**b**) Average Correlation between the blocks; (**c**) Average PLV in the blocks; (**d**) Average Correlation between the blocks.

Figure 4 shows the average correlations and PLV of the randomly selected samples. The vector $D^{T}vec\left(X^{T}\right)$ has the most intra-correlation structure in the blocks (over 1000 repetitions). We used Matlab version 8.5 on a PC with 12 GB RAM, and a 2.8 GHz Core i7 intel CPU. Our results agree with [19,20] that there exist non-linear dependency between the EEG channels and also within each channel. Unlike the correlation measure, the PLV is invariant to the type of vector (*i.e.*, it gives the same results for the $D^{T}vec\left[X^{T}\right],\;D^{T}vec\left[X\right]$, $D^{T}x_{l}$). Although the PLV shows a more dominant dependency, it is not meant to replace the correlation type dependency [20]. For this reason we have added the PLV measure in Equation (8) of the BSBL-BO algorithm so as to exploit the non-linear dependency in the EEG signals.

### 4.3. Error Metrics and Experiements

The original data are compressed at different compression rates given by $CR\%=\left(1-\frac{M}{N}\right)100$. Please note that the compression ratio $\frac{N}{M}=2:1$ corresponds to CR% = 50%. In our experiments we compress the EEG datasets at different rates including, 50, 60, 70, 80, and 90% compression rates. For these expriements, windows of 4 s each were randomly selected from the three datasets, 200 windows from each channel and for each subject. We measured the reconstruction quality using the Normalized Mean Square Error (NMSE). During the recording of the EEG datasets, a voltage DC bias was present in the electrodes. This bias could cause misleading results when calculating NMSE, to avoid this bias the mean of each signal is subtracted. For a fair evaluation we use the NMSE proposed in [6], $NMSE\left(x,\hat{x}\right)=\;\frac{\left\|X-\hat{X}\right\|}{\left\|X-\mu_{X}\right\|}$.

### 4.4. Compression/Decompresion Results

The compression was done using the sparse binary matrix as a sensing matrix. This matrix has only two non-zeros (*i.e.*, ones) in each column selected at random. The length of the matrix depends on M and this depends on the compression rate. For the MMV problem, our proposed BSBL-LNLD, and the BSBL-BO algorithims are applied. Based on Figure 4, the smaller the block size (*i.e.*, the more number of blocks per epoch), the higher the dependency measures. This however, causes a slow performance in Matlab 8.5. For this reason, a block size of 92 is chosen because it is found to be a suitable tradeoff.

**Figure 5:**
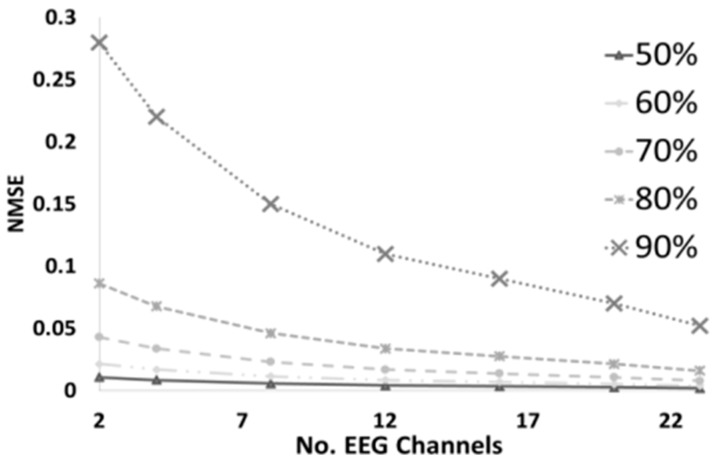
NMSE vs Number of Channels of proposed method at different compression % rates.

Figure 5 shows the performance of our proposed MMV method as the number of EEG channels increases. The more EEG channels (spatial data), the lower the reconstruction error. This is because the spatial dependence between channels increases as the number of channels increase. This promotes more joint dependency which makes the decompression more immune to compression rate values.

In our experiments we compared the performance of our proposed MMV method (BSBL-LNLD) with the following state of the art decompression EEG algorithms as:

(1) The tMFOCUSS proposed in [10] is a modified version of the MFOCUSS. It works by capturing the temporal correlation in the channels. The modification lies in replacing the norm minimization with the Mahalanobis distance.
(2) The TMSBL method proposed in [9]. It is a Bayesian approach. It defines the signal in the hyper-parameter space instead of the Euclidian space such as in *l1*/*l*2 minimization techniques. The hyper-parameter space is defined by temporal correlation and sparse modelling, this approach is called the Automatic Relevance Determination as proposed in [9,34]. By using Expected Maximization, the hyper-parameters are estimated from the posterior information, which is derived from a prior and the log-likelihood of the compressed signal. One can argue that TMSBL is similar to BSBL-BO in its basic approach and derivation. However, BSBL-BO reconstructs the signal in blocks form, unlike TMSBL.

(3) Recently, the BSBL-BO approach [1] was compared with the STSBL-EM algorithm presented in [12]. The comparison was performed on BCI data at different compression rates such as 50%, 60%, 70%, 80%, and 90%. It was shown that the decompression of SMV BSBL was less accurate than STSBL-EM. Two learning hyper-parameters were introduced in STSBL-EM, to capture the correlation between the blocks in the temporal and spatial domains. STSBL-EM learns the parameters by temporally whitening the model at first, and then the spatial hyper-parameter is learned and the signals are estimated. Then the signals are spatially whitened and then the temporal hyper-parameter and the signals are estimated. This process repeats until convergence within 2 to 3 s on average. The repetitive whiting of the model reduces the correlation in the signals which causes less redundancy during decompression, hence less correlation amongst the blocks. Our results in the Table 1 show that compared to the other methods STSBL-EM does not achieve low errors at high compression rates.

The DCT transform matrix is used for all experiments for all the algorithms, the proposed BSBL-LNLD, tMFOCUSS, TMSBL, STBSL, and BSBL-BO methods. These five methods were applied on the MMV problem. For the SVM problem only BSBL-LNLD and BSBL-BO, were applied as the others are only applicable to the MMV problem. Thus single channels are compressed channel by channel for the SMV problem, and multiple channels are compressed simultaneously in case of the MMV problem. For the SMV problem, the EEG data is compressed for each vector such that $y_{l}=Ax_{l},\forall l=\left[1,2,\;\;..,\;\;L\right]$. In case of the MMV problem, the EEG signals were compressed using the vector $y=Avec\left[X^{T}\right]$. As shown above the proposed method can sustain good recovery results even at high compression rates e.g., CR 90% (10:1 compression ratio). 

To the best of our knowledge, these are the best results that have been achieved so far with respect to obtaining high compression rates and low construction errors for the EEG signals in compressed sensing. Not ignoring that fact that the JPEG2000 still achieves the most accurate results at high compression rates, however, it is not suitable for WBANs.

**Table 1:** NMSE of the different methods.

CR		90%	85%	80%	70%	60%	50%
Compression Experiment							
**NMSE (BCI DataSet)**	BSBL-LNLD (Multichannel)	0.065	0.058	0.016	0.008	0.005	0.002
	BSBL-BO (Multichannel)	0.094	0.089	0.075	0.014	0.006	0.003
	BSBL-LNLD (SingleChannel)	0.461	0.384	0.242	0.154	0.094	0.045
	BSBL-BO (SingleChannel)	0.551	0.414	0.318	0.217	0.134	0.089
	STBSL-EM	0.791	0.427	0.133	0.038	0.017	0.009
	TMSBL	0.248	0.178	0.066	0.04	0.022	0.014
	tMFOCUS	0.665	0.269	0.077	0.035	0.018	0.011
**NMSE (Seizure DataSet)**	BSBL-LNLD (Multichannel)	0.242	0.191	0.174	0.114	0.097	0.035
	BSBL-BO (Multichannel)	0.311	0.257	0.216	0.165	0.114	0.058
	BSBL-LNLD (SingleChannel)	0.457	0.412	0.35	0.261	0.156	0.098
	BSBL-BO (SingleChannel)	0.671	0.575	0.472	0.319	0.228	0.147
	STBSL-EM	0.984	0.728	0.419	0.166	0.091	0.032
	TMSBL	0.698	0.687	0.217	0.154	0.11	0.036
	tMFOCUS	0.912	0.757	0.683	0.441	0.098	0.021
**NMSE (Sleep DataSet)**	BSBL-LNLD (Multichannel)	0.148	0.135	0.095	0.064	0.009	0.004
	BSBL-BO (Multichannel)	0.176	0.153	0.113	0.094	0.015	0.007
	BSBL-LNLD (SingleChannel)	0.388	0.265	0.147	0.092	0.058	0.029
	BSBL-BO (SingleChannel)	0.475	0.356	0.225	0.134	0.075	0.044
	STBSL-EM	0.89	0.561	0.315	0.126	0.065	0.007
	TMSBL	0.352	0.243	0.156	0.114	0.072	0.009
	tMFOCUS	0.864	0.587	0.413	0.324	0.054	0.017

### 4.5. Power Consumption Simulation

The power consumption evaluation of the sensor node is obtained using an open-source cycle-accurate Wireless Sensor Network (WSN) simulator called Avrora [35]. It emulates the sensor node circuitry by providing a virtual operating environment of the execution. An emulator software for an Atmel AVR micro-controller sensor platform, Mica2 and MicaZ, is provided in Avrora. It is used to provide a detailed monitoring evaluation of different behaviors such as packet transmission, energy monitoring, interrupts and stack usage. The energy monitor in Avrora provides the state changes of the sensor node and estimates the power consumption of each component of the sensor platform.

The compression techniques for the SMV and the MMV problem is implemented in Network Embedded Systems C for TinyOS [36]. TinyOS is an embedded operating system (OS) that provides hardware abstraction for operations such as packet transmission, storage and input/output (I/O). The code was compiled and simulated for a MicaZ sensor platform using Avrora. The MicaZ consists of an ATMega 128 micro-controller, ZigBee radio, 4 KB of RAM and 128 KB of flash memory. To simplify the evaluation process, real-time EEG acquisition simulation was not performed. Alternatively, a short epoch segment of EEG data was loaded into the memory to simulation for a 1 s data window. This simplification does not affect the evaluation results of the power consumption because the EEG sensing is the same across all epochs. The majority of the power on the sensor node is consumed by the micro-controller and the wireless transmitter. Approximately 20% of power consumption is accounted for the micro-controller, while 70% of the power is accounted for the radio transmitter [37]. For this reason usually the main focus of the power consumption is on the micro-controller and the wireless transmitter.

The results of the total power consumption on the sensor node is broken down into the code execution of the microcontroller, wireless transmitter radio, and flash memory. The power consumption of the sensor node of different CR% is estimated over a span of 1 hour of data which is sampled at 256 Hz. The battery life is estimated assuming that the battery used is a 3 Volt of 200 mAh. The battery life is estimated using the following equation: $BatteryLife=0.7\;\frac{Battery\;Capacity\;\left(mAh\right)}{Total\;load\;current\;/\;hour\;\left(mA\right)}$. The results is shown in the following Table 2.

**Table 2:** Breakdown of power consumption results at different compression rates in milliwatts.

CR	MCU	Transmitter	Memory	Total (mW)	Battery Life hrs (3V, 200 mAh)
0% (No Compression)	46.14	160.68	0	20.82	3.04
50%	20.07	30.67	13.60	64.34	9.79
60%	19.24	20.58	13.25	53.07	11.87
70%	18.41	14.1	12.91	45.42	13.87
80%	17.72	9.65	12.76	40.14	15.69
90%	17.04	6.67	12.78	36.49	17.27

## 5. Conclusions and Future Work

This paper proposes a novel method for compressing multi-channel EEG signals using compressive sensing. It introduces a way for representing the multi-channel EEG data measurements as a vector that has a significantly better “block” sparsity structure than the conventional one. The DCT coefficients of the reorganized vector is shown experimentally to have a high sparse and redundant block sparse structure. That is the new organization of the data results in a high number of sparse blocks. This enables the state-of art BSBL-BO to produce better reconstruction results of EEG data that have been compressed using compressive sampling. These results are then further improved by modifying the BSBL-BO method so that it exploits both the linear and non-linear dependencies in the EEG data and not only the linear one.

Previous works have shown that neurophysiological signals (including EEG signals) have linear and non-linear dependencies within each channel and between channels. To find the characteristics of the linear and non-linear dependencies in the proposed EEG vector representation, we experimentally calculate the average correlation and phase locking values of various EEG multi-channel data. To exploit these dependencies, we propose a modification to the parameters of BSBL-BO. The modified BSBL-BO is then applied on the sparse vector that results from our proposed representation of the EEG data. We show that these modifications enable BSBL-BO to exploit not only the linear dependency but also the non-linear ones. We also studied the characteristics of the reorganized DCT coefficients vector. We used correlated and uncorrelated random signals to prove that the sparse structure of the reorganized DCT coefficients is reproducible in correlated signals. The redundancy in the block structure increases with the increase in the number of correlated channels.

The proposed compressed sensing technique is applied on the MMV and the SMV problems. The compressed signals were decompressed using different existing algorithms for comparison. Two datasets of EEG signals of different patients and a third dataset of brain computer interface data were used. The results show that, the proposed BSBL-LNLD method results in a significant lower error compared to other methods, even at high compression rates such as 10:1. To the best of our knowledge, the results obtained are the best in the (WBAN) literature for EEG signals, JPEG2000 still remains the best compression technique in terms of accuracy at high compression rates, but it is not suitable for WBANs due to its high power consumption.

Mostly, the sensor architecture of a WBAN framework consists of a sensing part, processing and data transmission parts. Approximately 20% of power consumption is accounted for the micro-controller and the sensor, while 70% of the power is accounted for the radio transmitter [37]. The data transmission accounts for the most significant power consumption. That is the reason why reducing the power consumption of the data transmission has been an interesting topic in previous works. Therefore, it is essential to transmit fewer amounts of bits to achieve the lowest possible energy consumption. This is usually how the sensor’s battery lifetime is extended, provided that the recovery quality is high to achieve a reliable operation in WBAN applications. Only few papers shows little improvement to solve the power consumption of sensing, processing, and data transmission power all together [13,14,15]. Their work does not achieve high compression rates of the EEG, hence the recovery quality is too low which is reliable for WBAN applications. This work only solved the data compression problem achieving high recovery quality at high compression rates. However, it did not propose a solution for the power consumption of the sensing, processing, and data transmission together. In the future work, we intend to find a solution that accounts for high recovery quality during high data compression (10:1) while optimizing the sensing and processing power as well.
